# Care that Matters: Quality Measurement and Health Care

**DOI:** 10.1371/journal.pmed.1001902

**Published:** 2015-11-17

**Authors:** Barry G. Saver, Stephen A. Martin, Ronald N. Adler, Lucy M. Candib, Konstantinos E. Deligiannidis, Jeremy Golding, Daniel J. Mullin, Michele Roberts, Stefan Topolski

**Affiliations:** 1 Department of Family Medicine and Community Health, University of Massachusetts Medical School, Worcester, Massachusetts, United States of America; 2 Swedish Cherry Hill Family Medicine Residency, Seattle, Washington, United States of America; 3 Freelance Science Writer, Paxton, Massachusetts, United States of America

## Abstract

Barry Saver and colleagues caution against the use of process and performance metrics as health care quality measures in the United States.

**Summary Points**

- There is limited evidence that many “quality” measures—including those tied to incentives and those promoted by health insurers and governments—lead to improved health outcomes.
- Despite the lack of evidence, these measures and comparative “quality ratings” are used increasingly.
- These measures are often based on easily measured, intermediate endpoints such as risk-factor control or care processes, not on meaningful, patient-centered outcomes; their use interferes with individualized approaches to clinical complexity and may lead to gaming, overtesting, and overtreatment.
- Measures used for financial incentives and public reporting should meet higher standards.
- We propose a set of core principles for the implementation of quality measures with greater validity and utility.

Everyone aspires to quality health care. Defining “quality” seems straightforward, yet finding metrics to measure health care is difficult, a point recognized by Donabedian [1] almost half a century ago. While we often associate quality with price, Americans have the world’s most expensive health care system, yet have disappointing patient outcomes [2]. As a means to improve outcomes and control costs, payers in the United States, United Kingdom, and elsewhere are increasingly using metrics to rate providers and health care organizations as well as to structure payment.

Payers demand data in order to pay providers based on “performance,” but such quality measures and ratings are confusing to patients, employers, and providers [3,4]. Despite recent flaws in implementing measures for Accountable Care Organizations (ACOs), the Centers for Medicare and Medicaid Services (CMS), which administers national health care programs in the US, is moving towards linking 30% of Medicare reimbursements to the “quality or value” of providers’ services by the end of 2016 and 50% by the end of 2018 through alternative payment models [5]; more recently, CMS announced a goal of tying 85% of traditional fee-for-service payments to quality or value by 2016 and 90% by 2018 [6]. Earlier this year, the Medicare Payment Advisory Commission cautioned that “provider-level measurement activities are accelerating without regard to the costs or benefits of an ever-increasing number of measures” [7].

Evidence connecting many quality measures with improved health outcomes is modest, and metrics may be chosen because they are easy to measure rather than because they are evidence-based [8]. The Institute of Medicine (IOM) has warned against using easily obtained surrogate endpoints as quality indicators, because achieving them may not yield meaningful health outcomes [9]. When evidence does exist, the relationship between risk and biomarkers is typically continuous—yet measures often use discrete cutoffs. With payment at stake, clinicians and organizations may be tempted to game the system by devoting disproportionate effort to patients barely on the “wrong” side of a line rather than focusing on those at highest risk [10,11]. Some well-known quality measures do not perform as intended, or may even be associated with harm (e.g., drug treatment of mild hypertension in low-risk persons has not been shown to improve outcomes [the yet-to-be-published SPRINT trial enrolled high-risk participants]; glycemic control with drugs other than metformin in type 2 diabetes may cause harmful hypoglycemia yet fail to appreciably reduce morbidity and mortality) (Table 1 and S1 Table) [12–17]. This phenomenon is described as “virtual quality” [18]. Though some guidelines emphasize shared decision making [19], patient preferences are rarely addressed in guidelines.

**Table 1:** Ways targets distort care (see S1 Table for further detail and references).

Predictable Distortion	Example
Prioritizing easier care for healthier people	Focus on moving a healthy patient’s systolic blood pressure from 141 mm Hg to 139 mm Hg, thereby crossing the 140 mm Hg threshold, rather than help someone with a heart attack history improve from 180 mm Hg to 155 mm Hg.
Overtesting	Repeat colon cancer screening too early if prior test not billed for by current insurer; overuse of colonoscopy [20]. Frequent testing of low-density lipoprotein (LDL) or Hemoglobin (Hgb) A1c levels despite no known health benefit.
Distortion of informed consent	Test all age-eligible adolescents for chlamydia even if they deny sexual activity.
Overmedication	Use anti-hyperglycemic medications other than metformin to lower HgbA1c levels in type 2 diabetes, despite limited evidence of benefit and significant risk of harm [21,22].
Distraction from patients’ needs	Focus on surrogate markers rather than what is meaningful to the patient (which is likely not a performance measure).
Flawed sense of actual impact	Follow performance measure that is incentivized rather than a meaningful one, such as smoking cessation, which is not incentivized.
Privilege process rather than experience of care	Certify that a rushed well-child visit has happened, not that it was thoughtful, compassionate, effective, and meaningful.
Expansion of denominator of those who are considered “sick”	Include patients who marginally meet criteria for diabetes or hypertension, thus increasing the proportion of patients with mild, easily controlled disease and assuring that practices have greater proportions of patients who meet a performance measure.

Process and performance metrics are increasingly used as quality measures. One influential US program has stated that its “incentive payments are determined based on quality measures drawn from nationally accepted sets of measures” [23]. But these measures are typically derived from the Healthcare Effectiveness Data and Information Set (HEDIS), whose sponsor states they “were designed to assess measures for **comparison** among health care systems, not measures for **quality improvement**” (boldface in original) [24]. ACO quality measures not created by CMS carry the disclaimer that “These performance measures are not clinical guidelines and do not establish a standard of medical care, and have not been tested for all potential applications” [25]. When sponsors attach such disclaimers to their metrics, it is appropriate to question their use in public reporting and financial incentives. Limited understanding of the risks and benefits of testing, difficulties inherent in the communication of complex risk information, and misplacement of trust in advocacy organizations make physicians, patients, and payers susceptible to the erroneous impression that poorly chosen targets are valid and appropriate [26].

In our Massachusetts clinical practices, we have encountered examples of questionable targets across several organizations, such as the following: encouraging unnecessary urine microalbumin testing of diabetic patients already taking angiotensin-converting enzyme inhibitors or angiotensin receptor blockers [27], unnecessary fecal blood testing in patients who had undergone colonoscopy within the past 10 years (but not credited because the current insurance plan had not paid for it) [28], instructing women aged 40–49 years to schedule a mammogram rather than engaging them in shared decision making [29], and encouraging clinicians to start medications immediately for patients with diabetes whose blood pressure, low-density lipoprotein (LDL), or hemoglobin A1c is mildly elevated, rather than following recommendations for lifestyle interventions first [30–32]. Similarly, practitioners in the UK have received incentives for dementia screening, despite criticism that evidence to justify dementia case finding was lacking [33]. If non–evidence-based targets are created and supported with financial incentives, changes in patient care may be inappropriate (Table 1) [8,34].

Fee-for-service has been a critical driver of runaway costs and disappointing health outcomes in the US. “Quality” measures provide newer, different incentives, but may not achieve their purpose and may divert us from more thoughtful approaches and useful interventions, such as addressing social determinants, multimorbidity, and individualized care [35–39].

We believe there must be fundamental change in our approach to quality measurement. Surrogate and incompletely validated measures may, at times, appropriately inform quality improvement, but measures used for public reporting and financial incentives should meet higher standards. We propose an initial set of principles to guide the development of such quality measures (Box 1). Such measures should merit public trust, earn the support of clinicians, and promote the empowerment of patients. Their development should be open and transparent with careful attention to the best evidence of utility.

**Box 1. Core Principles for Development and Application of Health Care Quality Measures**

Quality measures must:

1. address clinically meaningful, patient-centered outcomes;
2. be developed transparently and be supported by robust scientific evidence linking them to improved health outcomes in varied settings;
3. include estimates, expressed in common metrics, of anticipated benefits and harms to the population to which they are applied;
4. balance the time and resources required to acquire and report data against the anticipated benefits of the metric;
5. be assessed and reported at appropriate levels; they should not be applied at the provider level when numbers are too small or when interventions to improve them require the action(s) of a system.

Quality improvement is a continuous process. Our principles were developed through consensus and are neither perfect nor exhaustive; like other guidelines, they will benefit from evaluation and modification. Good quality measures should inform consumers, providers, regulators, and others about the quality of care being provided in a setting, so the primary concerns for good measures are content validity and impact on health.

## Implications of the Guiding Principles

We are critical of current quality measures and believe many should be abandoned. In a 2013 US Senate hearing, a representative of business interests urged Medicare and other insurers to sharply reduce the number of indicators being used and “measure and report the outcomes that American families and employers care the most about—improvements in quality of life, functioning, and longevity” [40]. The Institute of Medicine’s 2015 report, *Vital Signs*: *Core Metrics for Health and Health Care Progress*, highlights how “many measures focus on narrow or technical aspects of health care processes, rather than on overall health system performance and health outcomes” and finds that the proliferation of measures “has begun to create serious problems for public health and for health care” [41,42].

Another recent editorial, highlighting the importance of intrinsic versus extrinsic motivation for physicians, asks: “How Did Health Care Get This So Wrong?” [43]. In the UK, a study looking at effects of the Quality and Outcomes Framework (QOF)—a broad-based array of metrics designed more systematically than similar efforts in the US—concluded that it appeared to have changed the nature of the consultation so that the biomedical agenda related to the QOF measures is prioritized and the patient’s agenda is unheard [44].

Patient-centeredness means that quality measures need to reflect outcomes experienced and valued by patients. Patient satisfaction is clearly a critical construct, but clinicians have a responsibility beyond maximizing satisfaction. Patient-reported outcomes, such as those being gathered by the US National Institutes of Health PROMIS program (www.nihpromis.org) and the International Consortium for Health Outcomes Measurement (ICHOM) [45], should also be included. In Table 2, we summarize some of the current, flawed approaches to quality measures and our recommendations for approaches consistent with our suggested guiding principles.

**Table 2:** Comparison of typical performance measures and author recommendations.

Current Approaches	Recommended Approaches
Binary (cut-point) thresholds of risk	Continuous measures of risk
Surrogate outcomes	Patient-centered outcomes
No accounting of staff effort required to impact performance measure	Accounting of staff effort
Lack of emphasis on shared decision-making and eliciting patient preferences	Individualization and shared decision-making as a default expectation
No articulation of NNT, NNH, NNS	Transparency and referencing of NNT, NNH, NNS
Measures focused on individual risk factors	Aggregate risk measures
Isolated morbidities	Recognition that multimorbidity may modify or invalidate some measures in individuals
No accounting for social determinants of health	Inclusion of social determinants of health
Multiple metric sources with varying biases and transparency	Single, independent, transparent, unbiased source

* NNT: number needed to treat; NNH: number needed to harm; NNS: number needed to screen

Our guiding principles (Box 1) first recognize that quality measures should reflect meaningful health outcomes. Surrogate measures do not satisfy this principle. Clinical trial results are often not attained in the real world, and there should be evidence that quality measures do, in fact, substantially improve health outcomes across various locales and practice settings. Verification will often be beyond the capacity of a single organization. Careful analysis of data from large populations will be required to ascertain whether the expected benefits are being produced.

Similarly, extrapolation from observational studies should not be used to determine quality measures—this often confuses treatment outcomes with natural risk factor distributions. Binary measures based on dichotomizing continuous risk factor measurements (e.g., blood pressure, hemoglobin A1c) should not be used. Aggregate risk measures, such as the Global Outcomes Score [10] will usually be preferable to individual risk factors. Quality improvement efforts based on aggregate measures are more likely to yield true health improvements and reduce gaming and other distortions in provider activity; aggregate measures are also more robust than isolated individual risk factors. To maximize use, aggregate measures should be available for use without charge [46]. One could argue either that, while mathematically complex, these measures should be open source to ensure fairness and transparency or, contrarily, that to minimize gaming/reverse engineering, they should be black boxes with trusted curators and frequent updating.

After years of application of “quality” measures tied to incentive payments, many clinicians have adopted practices to optimize revenue. Some of these practices are now out of step with current evidence. It is known that frontline clinicians are slow to integrate changes informed by recent evidence [47]; this problem is compounded when they are paid to do things “the old way.” It is therefore essential that quality measures evolve in a manner that demonstrates a timely response to new evidence; in this way, up-to-date quality measures can be a productive driver of early adoption of evidence-based interventions. The difficulty of changing established practices also highlights that high-stakes quality measures must be chosen carefully, focusing on important health outcomes and based on the most robust evidence.

Quality measures should reflect that a provider has elicited, explored, and honored patient values and preferences, and not merely indicate whether a test or intervention has been performed. To do otherwise strikes at the heart of patient-centered care. Because most health care interventions carry risk of causing harms, measures should reflect overutilization [16,48] as well as underutilization of care.

There should be an estimate of the expected magnitude of improved health from each quality measure. Clinical interventions to decrease smoking have a far greater impact on health than glycemic control for type 2 diabetes [49–51], yet current approaches obscure these relative contributions. Similarly, dental care [52] and effective treatment of alcohol [53] and opiate [54] addiction—highly meaningful to patients and strongly contributory to health (in terms of disability-adjusted life years)—are currently not quality measures. There should also be recognition of the potential for unintended downstream consequences, such as causing harm through interventions that may lead to overdiagnosis/overtreatment and through the associated opportunity costs [55].

We call for recognition that there are costs (time and resources) associated with acquiring and reporting data. The expected benefits of a measure must be weighed against direct and opportunity costs of data collection. Just as the Paperwork Reduction Act in the US requires an accounting of the time required for form completion, quality measures should include an estimate of the costs they will require. These costs may be mitigated to the extent that electronic health records can capture necessary data as part of the care process rather than data entry being required primarily for measurement.

Finally, there should be acknowledgement that improved health is often the result of actions by multiple parties at multiple levels, not individual providers. In many cases, patient action (or inaction) is critical and individual providers have limited influence [56]. Cancer screening or immunization typically is attributable to the influence of a larger system, not an individual clinician. It also means that data must be aggregated to a level where the numbers have statistical meaning, including understanding of signal-to-noise ratios. Also, social determinants of health are often more influential than individual clinicians. Communities or health care systems (e.g., ACOs) could be measured on their abilities to influence social determinants of health, such as food security, housing, etc. Altering these critical social determinants is more desirable than paternalistic or coercive influence through providers.

## Making Progress toward True Quality Measures

No simple strategy exists to move forward quickly, and we must guard against implementing flawed measures and then, years later, acknowledging their flaws. The best strategy for durability is likely one that uses the face validity of health—not surrogate outcomes—as the primary goal.

What do we know well enough to act upon? Our answers all come with caveats. Global risk measures are likely to be superior to individual risk factor measurements, but it is difficult to extrapolate from population findings to the individual. Patient satisfaction is an important, albeit incomplete, measure of quality. Many measures should be more preference-driven than we might like to admit, particularly for non-urgent decisions. In addition, asking health care providers to take responsibility for chronic conditions (e.g., hypertension, diabetes, obesity) over-emphasizes the role of the clinician. This is especially relevant when public health interventions that target social and environmental factors are much more likely to influence meaningful outcomes. We propose a set of performance measures that have higher face validity and better relationship to health (some examples are suggested in Box 2).

**Box 2. Examples of Patient-Centered Performance Measures**

1. Medication reconciliation in home after discharge
2. Home visits for indicated patients and coordinated care to meet their needs
3. Screening for and addressing fall risk
4. Patient self-assessment of health status (change over time)
5. Reduction of food insecurity
6. Ability to chew comfortably and effectively with dentition
7. Vision assessment and correction in place (e.g., patient has satisfactory glasses)
8. Hearing assessment and correction in place (e.g., patient has satisfactory hearing aids)
9. Reduction in tobacco use
10. Reliable access to home heating and cooling
11. Reliable transportation to appointments
12. Provision of effective contraception
13. Effective addiction care
14. Effective chronic pain care

Many groups have and will continue to produce metrics they label as “quality measures.” We propose, however, there should be an impartial curator of quality measures to be used for financial rewards or public reporting that should be financially and organizationally independent of potentially biasing groups. The US Preventive Services Task Force (USPSTF) provides a model for such an entity.

The American Association of Medical Colleges (AAMC) recently published a set of guiding principles for public reporting of provider performance [57], but they do not address the importance of patient-centeredness nor consideration of the potential for gaming. The Choosing Wisely campaign recognizes that quality is diminished when there is overtreatment, but selection of measures here, too, is problematic. Many quality measures will need to be based not on receipt of a test or a drug or pushing a risk factor below a target threshold but on facilitating an informed decision. Recent IOM recommendations for the development of guidelines provide a framework for developing quality measures [58].

We must not continue to mismeasure quality by prioritizing time- and cost-effectiveness over principles of patient-centeredness, evidence-based interventions, and transparency. Substantial resources are invested in public quality efforts that suggest progress, but implementing inappropriate measures is counterproductive, undermines the professionalism of dedicated clinicians, and erodes patient trust. The above principles are offered to help identify what is important for health, i.e., care that matters, so we may then develop quality measures more likely to reflect and enhance the quality of care provided, while minimizing opportunities for distortions such as gaming and avoiding the opportunity costs associated with efforts to optimize surrogate endpoints.

## Supporting Information

S1 Table Evidence for benefit and harm among selected patient populations and recent performance measures. (DOCX)

## Abbreviations

ACO: Accountable Care Organization
AAMC: American Association of Medical Colleges
CMS: Centers for Medicare and Medicaid Services; Hgb, Hemoglobin
IOM: Institute of Medicine
ICHOM: International Consortium for Health Outcomes Measurement
LDL: low-density lipoprotein
QOF: Quality and Outcomes Framework
USPSTF: US Preventive Services Task Force
