# Supplementary material for: Care that Matters: Quality Measurement and Health Care
Source: PLoS Med. 2015 Nov 17;12(11):e1001902. doi: 10.1371/journal.pmed.1001902 (PMC4648519; doi:10.1371/journal.pmed.1001902)
Supplement: S1 Table — (DOCX) [file pmed.1001902.s001.docx]

**Appendix A: Evidence for Benefit and Harm among Selected Patient Populations and Recent Performance Measures**

| **Patient Populations**  **and Performance Measures** | **Strong**  **Evidence for**  **Patient-Centered Benefit?^[[1]](#endnote-1)^** | **Strong**  **Evidence for**  **Patient-Centered**  **Harm?** | **Notes** |
| --- | --- | --- | --- |
| **Diabetes** (18 to 75 years old at the end of the year)  *Poor Control: Hemoglobin A_1_c > 9%*  *(lower score on the measure is better)* | N [1–3] | Y [4,5] | - For Type 2 Diabetes - “[T]he clearest indication for the use of antihyperglycaemic drugs in asymptomatic patients with type 2 diabetes remains the prevention of symptomatic hyperglycaemia” [6] - The newest American Diabetes Association guidelines emphasize a “patient-centered approach” and do not have specific HgbA1c targets [7] |
| **Depression** (18 and older)  *Proportion newly treated with an antidepressant medication who had effective acute phase treatment consisting of at least 84 days (12 weeks) of continuous treatment with antidepressant medication* | N [8] | Y [9] | - Does not specify severity of depression [8] - This measure does not consider alternative interventions that are as effective as pharmacological treatment [9] - The measure excludes patient choice to switch to another modality of effective therapy due to the side effects associated with pharmacological medications [9] |
| **Hypertension** (18 to 85 years old at the end of the year)  *Last result ≤139/≤89* | N [10]  (BP 140-159  /90-99) | Y [10] | - No benefit for pharmacotherapy in low-risk persons with mild hypertension [11] - Mild hypertension (BP 140-159/90-99) comprises approximately two-thirds of hypertension diagnoses [12] |
| **Breast Cancer Screening** (40-69 years at end of year)  *1 or more mammograms during the past 2 years* | N [13–16]  (ages 40-49) | Y [17–20] |  |
| **Cervical Cancer Screening** (21-64 years at end of year)  *1 or more Pap tests during the past three years* | Y | Y [21,22] | - Harm from overtesting prior to a 5 year interval for ages 30+ when cytology and HPV have been negative |
| **Chlamydia Screening** (female, 16-24 years old)  Sexually active and/or on contraception of any kind for any reason  *1 or more Chlamydia test during the year* | Y | Y [23,24] | - The Centers for Disease Control and Prevention report on Chlamydia screening emphasizes that “All culture and non‐culture tests may generate false positive and false negative results” and that “Clinicians need to be aware of the limitations of any test in low prevalence populations that they serve.” - The above holds true in a population that does not have a history of sexual activity as included in the metric (“on contraception of any kind for any reason”) |
| **Well Infant Care**  *Six (6) or more well child visits with a Primary Care Provider during the first 15 months of life*  **Well Child Visits 3-6 Year Olds**  *At least one comprehensive well-care visit with a primary care provider during the year*  **Well Adolescent Visits 12 – 21 Year Olds**  *At least one comprehensive well-care visit with a primary care provider or OB/GYN during the year* | N [25–28] | N [25–28] | - Moyer et al have found harms from certain behavioral modification interventions, false positive results, and the displacement of more effective interventions by less effective or ineffective ones [26] |

|  | No evidence of benefit or evidence of harm |
| --- | --- |
|  | Unclear evidence |
|  | Evidence of benefit |

1. Boussageon R, Bejan-Angoulvant T, Saadatian-Elahi M, Lafont S, Bergeonneau C, Kassaï B, et al. Effect of intensive glucose lowering treatment on all cause mortality, cardiovascular death, and microvascular events in type 2 diabetes: meta-analysis of randomised controlled trials. BMJ. 2011;343: d4169.

2. Gale EA. Is type 2 diabetes a category error? Lancet. Elsevier Ltd; 2013;381: 1956–1957. doi:10.1016/S0140-6736(12)62207-7

3. Romeo GR, Abrahamson MJ. The 2015 Standards for Diabetes Care: Maintaining a Patient-Centered Approach. Ann Intern Med. 2015; doi:10.7326/M15-0385

4. Pogach L, Aron D. The other side of quality improvement in diabetes for seniors: a proposal for an overtreatment glycemic measure. Arch Intern Med. 2012;172: 1510–2. doi:10.1001/archinternmed.2012.4392

5. Lipska KJ, Montori VM. Glucose control in older adults with diabetes mellitus--more harm than good? JAMA Intern Med. 2013;173: 1306–7. doi:10.1001/jamainternmed.2013.6189

6. Montori VM. Helping patients make sense of the risks of taking GLP-1 agonists. BMJ. 2013;346: f3692.

7. Inzucchi SE, Bergenstal RM, Buse JB, Diamant M, Ferrannini E, Nauck M, et al. Management of Hyperglycemia in Type 2 Diabetes, 2015: A Patient-Centered Approach: Update to a Position Statement of the American Diabetes Association and the European Association for the Study of Diabetes. Diabetes Care. 2014;38: 140–149. doi:10.2337/dc14-2441

8. Kirsch I, Deacon BJ, Huedo-Medina TB, Scoboria A, Moore TJ, Johnson BT. Initial severity and antidepressant benefits: a meta-analysis of data submitted to the Food and Drug Administration. PLoS Med. 2008;5: e45. doi:10.1371/journal.pmed.0050045

9. Performance Measurement Committee of the American College of Physicians. Diagnosis and Treatment of Depression: Review of the Performance Measures by the Performance Measurement Committee of the American College of Physicians [Internet]. 2015 [cited 4 Oct 2015]. Available: https://www.acponline.org/clinical_information/performance_measurement/measures/pmc_measure_review_depression.pdf

10. Diao D, Wright JM, Cundiff DK, Gueyffier F. Pharmacotherapy for mild hypertension. Cochrane Database Syst Rev. 2012/08/17 ed. 2012;8: CD006742. doi:10.1002/14651858.CD006742.pub2

11. Heath I. Waste and harm in the treatment of mild hypertension. JAMA Intern Med. 2013;173: 956–7. doi:10.1001/jamainternmed.2013.970

12. QuickStats: Percentage Distribution of Blood Pressure Categories Among Adults Aged >18 Years, by Race/Ethnicity --- National Health and Nutrition Examination Survey, United States, 1999--2004. MMWR Morb Mortal Wkly Rep. 2007;56: 611.

13. Bleyer A, Welch HG. Effect of three decades of screening mammography on breast-cancer incidence. N Engl J Med. 2012;367: 1998–2005. doi:10.1056/NEJMoa1206809

14. Gøtzsche PC, Jørgensen KJ. Screening for breast cancer with mammography. Cochrane Database Syst Rev. 2013;6: CD001877. doi:10.1002/14651858.CD001877.pub5

15. U.S. Preventive Services Task Force. Screening for breast cancer: U.S. Preventive Services Task Force recommendation statement. Ann Intern Med. 2009;151: 716–26, W–236. doi:10.1059/0003-4819-151-10-200911170-00008

16. Miller ABB, Wall C, Baines CJJ, Sun P, To T, Narod SA a. Twenty five year follow-up for breast cancer incidence and mortality of the Canadian National Breast Screening Study: randomised screening trial. BMJ. 2014;348: g366. doi:10.1136/bmj.g366

17. The benefits and harms of breast cancer screening: an independent review. Lancet. 2012;380: 1778–86. doi:10.1016/S0140-6736(12)61611-0

18. Moynihan R, Doust J, Henry D. Preventing overdiagnosis: how to stop harming the healthy. BMJ. 2012;344: e3502.

19. Ahmed H, Naik G, Willoughby H, Edwards AGK. Communicating risk. BMJ. 2012;344: e3996.

20. Kirwan CC. Breast cancer screening: what does the future hold? BMJ. 2013;346: f87.

21. Moyer VA. Screening for cervical cancer: U.S. Preventive Services Task Force recommendation statement. Ann Intern Med. 2012;156: 880–91, W312. doi:10.7326/0003-4819-156-12-201206190-00424

22. Kizer N, Peipert JF. Cervical cancer screening: primum non nocere. Ann Intern Med. 2012;156: 896–7, W315. doi:10.7326/0003-4819-156-12-201206190-00425

23. Low N, McCarthy A, Macleod J, Salisbury C, Campbell R, Roberts TE, et al. Epidemiological, social, diagnostic and economic evaluation of population screening for genital chlamydial infection. Health Technol Assess. 2007;11: iii–iv, ix–xii, 1–165.

24. Association of Public Health Laboratories and the Centers for Disease Control and Prevention. Laboratory Diagnostic Testing for Chlamydia trachomatis and Neisseria gonorrhoeae: Expert Consultation Meeting Summary Report. 2009.

25. Needlman R. What do we do with our 15 minutes? Pediatrics. 2012;130: e683–4. doi:10.1542/peds.2012-1519

26. Moyer V a, Butler M. Gaps in the evidence for well-child care: a challenge to our profession. Pediatrics. 2004;114: 1511–21. doi:10.1542/peds.2004-1076

27. Dinkevich E, Hupert J, Moyer VA. Evidence based well child care. BMJ. 2001;323: 846–9.

28. Schor EL. Rethinking Well-Child Care. Pediatrics. 2004;114: 210–216. doi:10.1542/peds.114.1.210

1. In determining the strength of evidence, we used the GRADE system for strength of recommendations. Guyatt GH, Oxman AD, Vist GE, et al. GRADE: an emerging consensus on rating quality of evidence and strength of recommendations. *BMJ (Clinical research ed.)*. 2008;336(7650):924–6. doi:10.1136/bmj.39489.470347.AD. [↑](#endnote-ref-1)
